# Central Effects of the Designer Drug Mephedrone in Mice—Basic Studies

**DOI:** 10.3390/brainsci12020189

**Published:** 2022-01-30

**Authors:** Anna Serefko, Gabriela Bielecka-Papierz, Sylwia Talarek, Aleksandra Szopa, Piotr Skałecki, Bernadeta Szewczyk, Maria Radziwoń-Zaleska, Ewa Poleszak

**Affiliations:** 1 Laboratory of Preclinical Testing, Chair and Department of Applied and Social Pharmacy, Medical University of Lublin, 1 Chodźki Street, 20-093 Lublin, Poland; aleksandra.szopa@umlub.pl; 2 Chair and Department of Applied and Social Pharmacy, Medical University of Lublin, 1 Chodźki Street, 20-093 Lublin, Poland; 3 Department of Pharmacology and Pharmacodynamics, Medical University of Lublin, 4a Chodźki Street, 20-093 Lublin, Poland; talareks@poczta.onet.pl; 4 Department of Commodity Science and Processing of Raw Animal Materials, University of Life Sciences, 13 Akademicka Street, 20-950 Lublin, Poland; piotr.skalecki@up.lublin.pl; 5 Department of Neurobiology, Maj Institute of Pharmacology, Polish Academy of Sciences, 12 Smętna Street, 31-343 Kraków, Poland; szewczyk@if-pan.krakow.pl; 6 Department of Psychiatry, Medical University of Warsaw, 00-665 Warsaw, Poland; maria.radziwon@wum.edu.pl

**Keywords:** mephedrone, rotarod test, chimney test, tolerance, elevated plus maze, pentylenetetrazol seizure tests, novel object recognition

## Abstract

Mephedrone belongs to the “party drugs” thanks to its psychostimulant effects, similar to the ones observed after amphetamines. Though mephedrone is used worldwide by humans and in laboratory animals, not all properties of this drug have been discovered yet. Therefore, the main aim of this study was to expand the knowledge about mephedrone’s activity in living organisms. A set of behavioral tests (i.e., measurement of the spontaneous locomotor activity, rotarod, chimney, elevated plus maze with its modification, novel object recognition, and pentylenetetrazol seizure tests) were carried out in male albino Swiss mice. Different dose ranges of mephedrone (0.05–5 mg/kg) were administered. We demonstrated that mephedrone at a dose of 5 mg/kg rapidly increased the spontaneous locomotor activity of the tested mice and its repeated administration led to the development of tolerance to these effects. Mephedrone showed the anxiolytic-like potential and improved spatial memory, but it did not affect recognition memory. Moreover, the drug seemed not to have any anticonvulsant or proconvulsant activity. In conclusion, mephedrone induces many central effects. It easily crosses the blood-brain barrier and peaks in the brain quickly after exposure. Our experiment on inducing a hyperlocomotion effect showed that mephedrone‘s effects are transient and lasted for a relatively short time.

## 1. Introduction

Mephedrone is one of the most popular synthetic cathinone derivatives used recreationally. Though it was synthesized in 1929, the drug did not receive much interest as a psychoactive agent for many years. After being introduced to the European market ca. 2007, mephedrone was quickly associated with the development of serious adverse reactions, including fatal intoxications, which raised concerns about the safety profile of this cathinone. In consequence, the substance was banned in the European Union countries and in several other non-European ones, including Israel, Australia, or the USA. However, it is still illegally sold under different street names (e.g., “meow meow”, “bubbles”, “M-CAT”, “cristal bath”, “kati”, “mephisto”) in the form of crystals, powder, capsules, or tablets, and it is used predominantly by oral ingestion and nasal insufflation. However, due to the high solubility of mephedrone powder in water, the drug is also administered via intramuscular or intravenous injection and rectal insertion [1,2].

Mephedrone belongs to the “party drugs” thanks to its psychostimulant and empathogenic effects [3], similar (though milder) to the ones observed after administration of cocaine, methamphetamine, and/or 3,4-methylenedioxymethamphetamine (MDMA). In fact, mephedrone is structurally similar to substituted amphetamines, which is why it shares some of their molecular and neurobehavioral effects [4,5]. Motbey and colleagues [6] observed the mephedrone-induced elevation in Fos expression in the cortex, striatum, ventral tegmental area, and supraoptic nucleus. Activation of these brain regions is also observed after exposure to methamphetamine and/or MDMA. Enjoyable and rapid but short-lived feeling of excitement, euphoria, urge to talk, reduced hostility, enhanced music appreciation, increased libido, improved alertness, and concentration have been reported by mephedrone users [7,8,9] since the drug easily crosses the blood-brain barrier [10,11]. These positive effects usually appear 15–45 min after oral administration of mephedrone (or a few min after nasal insufflations), and they last only ca. 2–3 h, which encourage mephedrone users to repeat the dose [12]. Therefore, even though a typical mephedrone dose is about 100–200 mg, an individual may consume it even up to 4 g during one session [7,13]. Positive effects of mephedrone’s action may be accompanied by negative immediate responses to this drug, such as pupil dilation, blurred vision, time distortions, or hot flushes [1]. Prolonged mephedrone use may result in psychological (including depression, panic attacks, agitation, paranoia, auditory and visual hallucinations, self-mutilation), renal (kidney injury), musculoskeletal (bruxism), and cardiovascular (i.e., hypertension, tachycardia, palpitations) adverse reactions [14,15]. The mechanism of action of mephedrone has not been fully described yet. However, most probably, mephedrone’s activity is attributed to its interaction with three major monoamine transporters (i.e., serotonin transporter SERT, dopamine transporter DAT, and norepinephrine transporter NET), serotonin 5-HT_1A_ and 5-HT2_A_ receptors, omega-1 receptors, as well as with α_1_- and α_2_-adrenoreceptors [10,16]. Several independent studies have demonstrated that mephedrone acts as a substrate of SERT, DAT, and NET induces the release of respective monoamines into the synaptic cleft, and blocks reuptake of these neurotransmitters [11,17,18,19,20,21,22,23]. There are few papers reporting the effects of mephedrone on the brain dopaminergic nuclei. Mentioned above Motbey et al. [6] showed an activation of the caudate-putamen and the ventral tegmental area (VTA) after administration of mephedrone in adolescent Wistar rats. Mephedrone also exerted a stimulatory effect on the nucleus accumbens (core and shell). Other studies demonstrated elevated dopamine and/or serotonin levels in the nucellus accumbens in adolescent and adult rats after exposure to different doses of mephedrone [17,20,24,25]. Furthermore, Grochecki et al. [26] showed a range of changes in the hippocampus of adolescent rats that can be detected after administration of mephedrone to adolescent rats, including elevated levels of matrix metalloprotease (MMP)-9, up-regulation of the NMDA receptor subunit GluN2B, and decreased levels of postsynaptic density protein (PSD)-95 levels. Naseri et al. [27] demonstrated hippocampal damage (i.e., an inhibition in cell growth and intensification of apoptosis) in offspring of female mice that were administered mephedrone during pregnancy. Though Angoa-Pérez and colleagues [28] did not observe any impairments in hippocampal serotonin nerve endings in female mice given mephedrone, in experiments by Martinez-Clemente et al. [29], administration of this drug induced a substantial loss of serotonin transporters in the hippocampus. The difference between these two studies could be due to differences in study design, particularly in tested doses, i.e., the latter research team used a significantly higher dose of mephedrone.

A number of preclinical studies revealed that mephedrone is also able to change animals behavior in many ways: it induces hyperactivity [30], antidepressant-like [31], or pro-depressive [29] effects measured by the forced swim test and/or the tail suspension test [32], excessive salivation [33], or repeated self-licking that may be followed by aggressive behavior and self-injuries [29]. Mephedrone can improve visuo-spatial associative memory and learning [34], but it may impair the working memory [35] and reference memory [36]. Moreover, it is known to disturb thermoregulation [10,18,37,38], temporarily diminish weight gain [26], induce relatively long-term alterations in the gut microbiome [5], reduce social preference ([39]; which is surprising given its pro-social effects in humans), display thermal antinociception [32], and to generate behavioral sensitization in relation to the repetitive movements [40] and to the total locomotor activity [30,41]. Similar to cocaine and amphetamines, this drug displays rewarding properties since it is readily self-administered by rats [18], facilitates intracranial self-stimulation in mice [42], and evokes conditioned place preference in rodents and planarians [41,43]. These behavioral findings correlate with the risk of addiction to mephedrone and its abuse in humans. It has been reported that chronic use of mephedrone may result in tolerance and/or abstinence syndrome [44]. However, Muskiewicz et al. [38] reported that mephedrone poses significantly less risk of overdose in comparison to methamphetamine or MDMA. Moreover, this synthetic cathinone does not induce higher lethality than the latter two drugs. It has been estimated that LD50 for mephedrone is 118.8 mg/kg (when assessed for mephedrone as the base) or 143.2 mg/kg (when assessed for mephedrone as the hydrochloride salt) in C57Bl/6J mice.

Though mephedrone is used worldwide both by humans (illegally) and in laboratory animals (legally), not all properties of this drug have been discovered yet. In the available literature, there are not many reports from behavioral experiments related to the central effects of mephedrone. Moreover, many discrepancies are found between the outcomes described in published manuscripts (resulting from, for example, diverse experiment schemes and equipment, laboratory conditions, applied animal strains), which suggest that further research is needed. Therefore, when designing the present study, we wanted to contribute to general knowledge about mephedrone’s activity in living organisms and to demonstrate some additional properties of this agent. We subjected adult albino Swiss mice to a set of behavioral tests: (1) measurement of the spontaneous locomotor activity (in order to check a time frame of the mephedrone-induced ambulatory hyperactivity), (2) rotarod and chimney tests (in order to investigate the impact of mephedrone on motor coordination and muscle relaxation), (3) elevated plus maze (EPM) test (in order to check an anxiolytic/anxiogenic potential of mephedrone), (4) modified EPM (mEPM) and novel object recognition (NOR) tests (in order to evaluate mephedrone effects on various aspects of learning and memory), and (5) pentylenetetrazol (PTZ) seizure test (in order to assess an anti-seizure/pro-seizure potential of mephedrone). We believe that outcomes obtained in the present study will allow us to better understand the biological effects of mephedrone.

## 2. Materials and Methods

### 2.1. Animals

Male albino Swiss mice (Farm of Laboratory Animals, Warsaw, Poland) that had an initial weight of 20–25 g were used. Animals were housed in groups of 10 and maintained at a temperature of 21 ± 1 °C and under a 12 h light/dark cycle. They had free access to standard food (Agropol, Marynin, Poland) and tap water. Each experimental group consisted of 8–10 animals. Mice were used only once. On the experimental day, i.e., after one week of adaptation and handling, the animals were randomly allocated to treatment groups. For the rotarod test and the chimney test, animals were randomly allocated to treatment groups after the final training session. The allocation sequence was concealed from a person who allocated animals (allocation concealment). Investigators were blinded. The inter-individual variations were minimized by purchasing animals that were similar in age and weight. All behavioral experiments were performed according to the binding European and local law related to experimentation on animals and were approved by the local ethics committee.

### 2.2. Drugs

Mephedrone (((RS)-2-methylamino-1-(4-methylphenyl) propan-1-one; Toronto Research Chemicals Inc., Toronto, ON, Canada) and pentylenetetrazole (Aldrich, Baden-Württemberg, Germany) were dissolved in saline (0.9% sodium chloride) ex tempore before experiments. Mephedrone was administered intraperitoneally (i.p.), whereas pentylenetetrazole was given subcutaneously (s.c.) at a volume of 10 mL/kg. Control animals were injected with the same volume of saline, either i.p. and/or s.c., depending on the study design. The doses of mephedrone were chosen on the basis of the literature data [31,41] and our preliminary studies.

### 2.3. Behavioral Tests

#### 2.3.1. Locomotor Activity Measurement

The locomotor activity of each mouse was recorded using a photocell apparatus (round Plexiglas cage, 32 cm in diameter, Multiserv, Lublin, Poland) which was placed in a sound-attenuated experimental room. Animals were placed into individual cages, 20 min after i.p. injection of mephedrone (0.05, 0.125, 0.25, 0.5, 1, 2.5 or 5 mg/kg) or saline (Experiment 1) or immediately after i.p. injection of mephedrone (5 mg/kg) or saline (Experiment 2). Locomotor activity was assessed as a number of photocell interruptions during a period of 10 and 30 min (Experiment 1) or 60 min (Experiment 2).

In order to check whether a repeated administration of mephedrone will result in the development of tolerance to its effect on the spontaneous locomotor activity in mice. A total of 5 mg/kg of this agent or saline were given i.p. once a day for 7 consecutive days. The spontaneous locomotor activity of animals was recorded 20 min after mephedrone/saline administration on the 1st, 2nd, 3rd, 5th and 7th day of the experiment. The assessment was carried out for 10 and 30 min. We defined tolerance as a statistically significant reduction in behavioral response compared to day 1 of treatment. To see whether the withdrawal effect will develop after discontinuation of mephedrone treatment (e.g., an increased locomotor activity), on the 9th day of the study, both groups of animals received only saline, and their locomotor activity was assessed for 10 and 30 min.

#### 2.3.2. Rotarod Test

The rotarod test was carried out as described before [45]. Mice were trained daily for 3 days on a bar (2 cm in diameter) rotating at a constant speed of 18 rpm (Ataner Zakład Usługowy Elektroniki Inż. K. Fic, Lublin, Poland). During each training session, mice were placed on a rotating rod for 3 min with an unlimited number of trials. The experimental session was conducted at least 24 h after the final training session, and only those animals were approved to the experimental session that were able to stay on the rotating rod for 60 s. Mice were placed on a rotating rod 20 min after mephedrone (0.125, 0.25, 0.5 or 1 mg/kg) or saline i.p. injection. The experimental session lasted for 60 s. The time spent on the rotating rod by a given mouse was measured.

#### 2.3.3. Chimney Test

The chimney test was carried out as described before [45]. Animals had to climb backward up a plastic tube (3 cm in inner diameter, 25 cm long). Mice were trained once a day for 3 days. The experimental session was conducted at least 24 h after the final training session, and only those animals were approved to the experimental session that were able to leave the chimney without much problem (up to 15 s). Mice were placed in the chimney 20 min after mephedrone (0.125, 0.25, 0.5 or 1 mg/kg) or saline i.p. injection. The experimental session lasted for 60 s. The time spent in the chimney by a given mouse was measured.

#### 2.3.4. Elevated plus Maze (EPM) Test

The EMP test was carried out as described before [46]. The experimental apparatus was a black Plexiglas maze with a central platform (5 × 5 cm), two open arms (30 × 5 cm), and two equal-sized enclosed (30 × 5 × 15 cm) arms opposite to each other. The maze was elevated to a height of 50 cm above the floor and illuminated by a dim light. A total of 20 min after mephedrone (0.05, 0.125, 0.25, 0.5, 1 or 2 mg/kg) or saline i.p. injection, a given mouse was placed in the central zone, facing the closed arm, and it was allowed to move freely around the maze for 5 min. The session was recorded by a video camera. The number of entries into open arms and the time spent in open arms were measured as indicators of the anxiogenic/anxiolytic-like potential, whereas the overall motor activity of animals in the EPM was measured by a number of entries into closed arms and by the total number of entries into either type of arms. After each mouse, to avoid confounding olfactory cues, the arms were cleaned with 70% ethanol and dried with paper towels.

#### 2.3.5. Modified Elevated Plus-Maze (mEPM) Test

The mEPM test was carried out as described before [47]. The experimental apparatus was a dark Plexiglas maze elevated to the height of 50 cm above the floor. It was shaped like a “plus” sign, and it consisted of a central platform (5 × 5 cm), two open arms (5 × 30 cm), and two enclosed arms (5 × 30 × 15 cm), which were opposite to each other. The mEPM test was conducted under red, dim light. A total of 20 min after mephedrone (0.125, 0.25, 0.5 or1 mg/kg) or saline i.p. injection, a given mouse was placed in the distal end of an open arm, facing from the central platform (the acquisition session). The time it took for the animal to reach one of the enclosed arms (transfer latency, TL1) was measured. When the mouse did not enter an enclosed arm within 90 s, it was placed in an enclosed arm, and it was allowed to explore the maze for an additional 60 s. In such cases, the TL1 value was recorded as 90 s. A total of 24 h later, the retention session (i.e., test trial) was carried out in the same manner as the acquisition session, but without injection of mephedrone and saline. The time it took for an animal to reach one of the enclosed arms (TL2) was measured. After each mouse, to avoid confounding olfactory cues, the maze was cleaned with 70% ethanol and dried with paper towels.

#### 2.3.6. Novel Object Recognition (NOR) Test

The NOR test was carried out as described before [48,49]. The experimental apparatus consisted of an open field box (36 × 30 × 26 cm) illuminated by a 25 W bulb, and it was located in a quiet room. For habituation, which lasted for 2 consecutive days, each mouse was placed individually into the open field box, and it was allowed to explore the box freely for 10 min. No objects were placed in the box during the habituation sessions. The day after habituation, the acquisition trial and the test trial occurred. In the acquisition trial, a given mouse was placed in the middle of the open field box, which contained two identical objects. The mouse was allowed to explore the two objects for 10 min. A total of 1 h later, the trial session was carried out. In the trial session, the mouse was put back into the open field box, but this time one of the objects used in the acquisition phase had been replaced by a novel one (with a different texture, color, and shape). Once again, the animal was allowed to explore the objects freely for 5 min, and the time spent exploring the familiar object (T_familiar_) and the time spent exploring the novel object (T_novel_), i.e., sniffing or touching an object with the nose and/or forepaws, were measured. After that, the preference index (PI) was calculated as follows: PI (100%) = (T_novel_ × 100%)/(T_familiar_ + T_novel_). Mephedrone (0.125, 0.25, 0.5 or 1 mg/kg) or saline were administered i.p. 20 min before the acquisition trial.

#### 2.3.7. Pentylenetetrazole (PTZ)-Induced Seizures

The PTZ animal model of epilepsy was induced as described before [50,51]. Mephedrone (1.25, 2.5, 5 mg/kg) or saline were administered i.p. 20 min before the s.c. injection of PTZ (110 mg/kg). Immediately after treatment with PTZ, animals were transferred to an open field (50 cm in diameter), and they were observed for 60 min for the incidents of clonic seizures, tonic convulsions, and death.

### 2.4. Statistical Analysis

Statistical analysis was carried out either by one-way ANOVA or two-way ANOVA followed by the Tukey’s, Dunnett’s, Kruskal–Wallis’s, or Bonferroni’s post-hoc test, depending on the study design. Differences between compared groups were considered significant when *p* was lower than 0.05. Statistical analyses and all figures were prepared using GraphPad Prism version 6.00 for Windows, GraphPad Software (San Diego, CA, USA).

## 3. Results

### 3.1. Effects of Mephedrone on the Spontaneous Locomotor Activity in Mice

**Figure 1:**
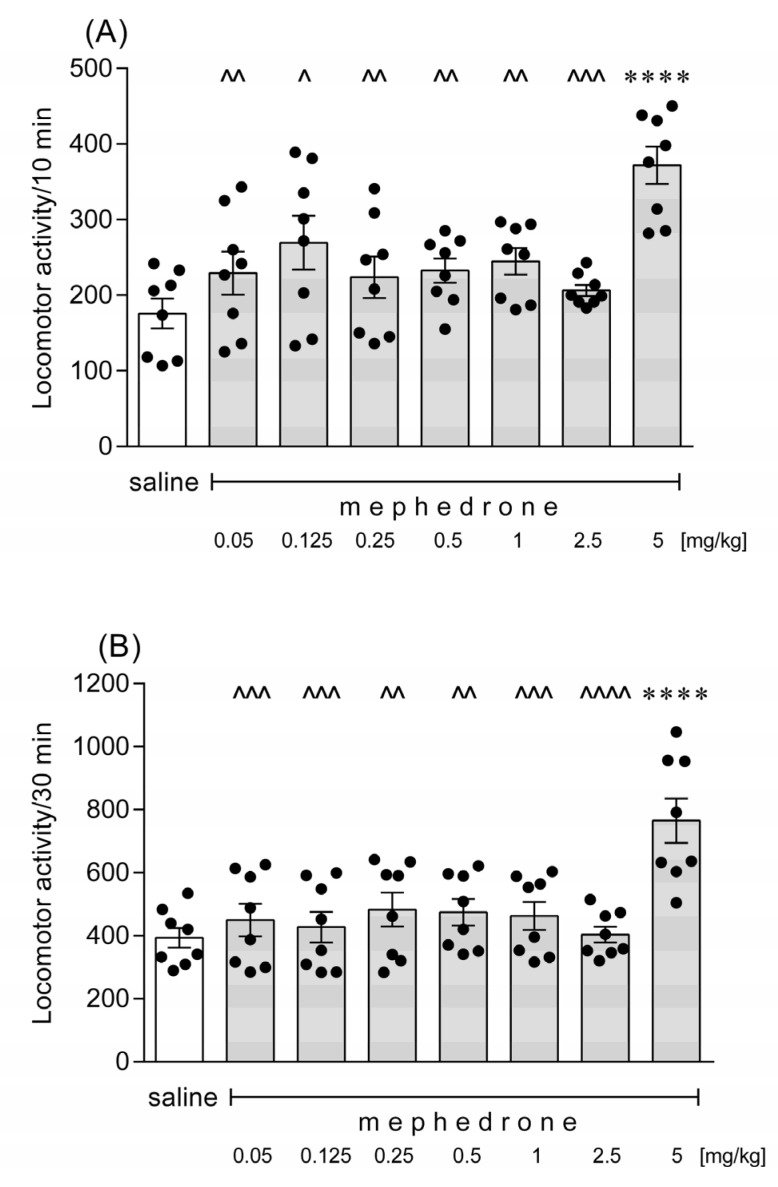
Effects of an acute administration of mephedrone on the spontaneous locomotor activity in mice. A single dose of mephedrone (0.05, 0.125, 0.25, 0.5, 1, 2.5 or 5 mg/kg) or saline was given intraperitoneally and 20 min after the injection the locomotor activity of animals was measured for (**A**) 10 min or (**B**) 30 min. The values represent mean ± SEM (*n* = 8 mice per group). **** *p* < 0.0001 versus saline; ^^^^ *p* < 0.0001, ^^^ *p* < 0.001, ^^ *p* < 0.01, ^ *p* < 0.05 versus mephedrone 5 mg/kg (Tukey’s post-hoc test).

Mephedrone given at a dose of 5 mg/kg increased the spontaneous locomotor activity of the tested mice as compared to the saline-treated group (*p* < 0.001), independently of the fact whether the assessment was carried out for 10 or for 30 min (Figure 1, Experiment 1). The lower doses of mephedrone (i.e., 0.05–2.5 mg/kg) did not influence the spontaneous locomotor activity of animals (*p* > 0.05). One-way ANOVA demonstrated significant differences between the groups: (A) F(7,56) = 6.109; *p* < 0.0001 for the 10 min measurement, and (B) F(7,56) = 6.208; *p* < 0.0001 for the 30 min measurement.

**Figure 2:**
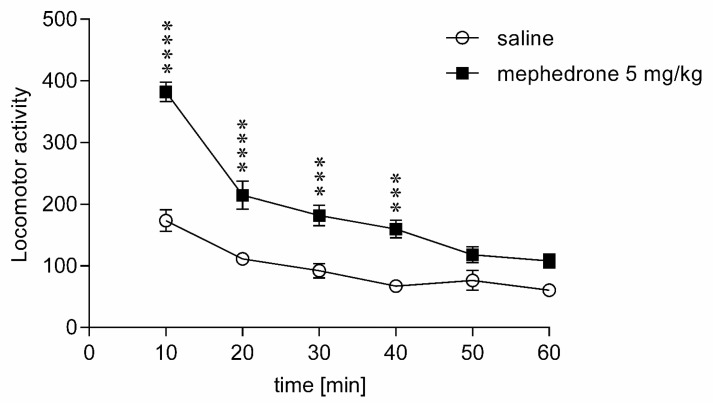
Time frame of the mephedrone influence on the spontaneous activity in mice. A single dose of mephedrone (5 mg/kg) or saline was given intraperitoneally, and immediately after the injection, the locomotor activity of animals was measured for 60 min. The values represent mean ± SEM (*n* = 8 animals per group). **** *p* < 0.0001, *** *p* < 0.001 versus saline (Dunnett’s post-hoc test).

Further experiments revealed that the mephedrone-induced hyperactivity in mice can be detected in less than 10 min after exposure to the active dose of mephedrone (5 mg/kg) and that it lasted for at least 40 min (Figure 2, Experiment 2). The spontaneous locomotor activity of the tested mice was normalized 50 min after the injection. Two-way ANOVA revealed a significant time-treatment interaction [F(5,84) = 8.80; *p* < 0.0001] with a significant effect of mephedrone treatment [F(1,84) = 137.61; *p* < 0.0001] and a significant effect of time [F(5,84) = 48.60; *p* < 0.0001].

### 3.2. Tolerance to the Mephedrone-Induced Hyperlocomotion in Mice

**Figure 3:**
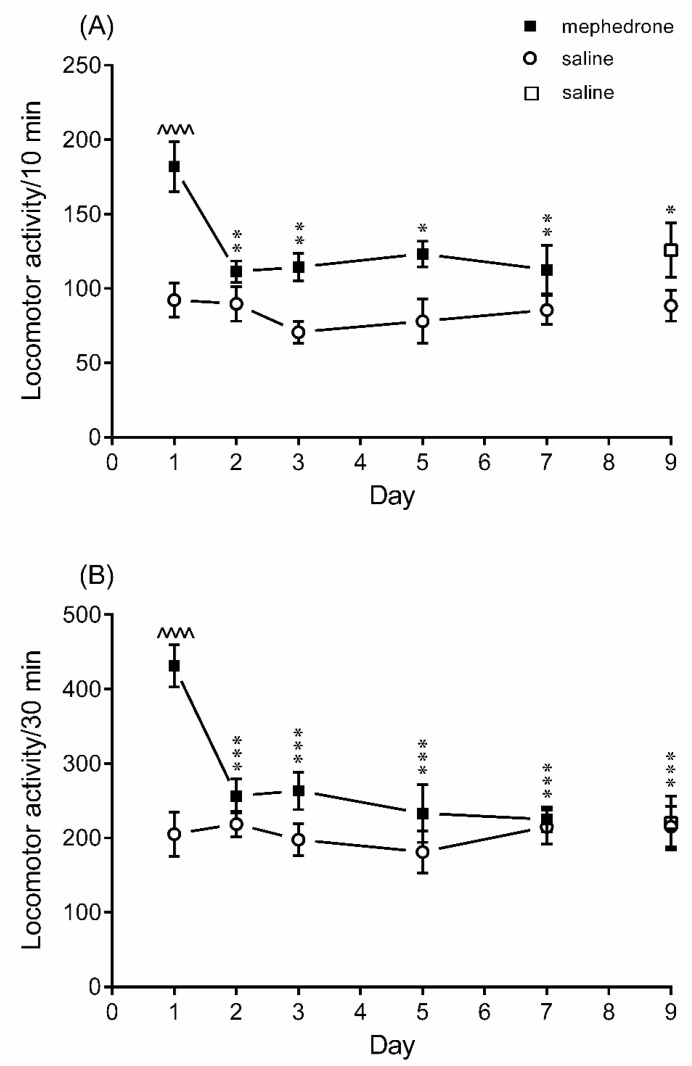
Development of tolerance to the mephedrone-induced effects on the spontaneous locomotor activity in mice. Mephedrone (5 mg/kg) or saline was given intraperitoneally once a day for 7 consecutive days, and on the 9th day, both groups of animals received only saline. The spontaneous locomotor activity of mice was recorded 20 min after the injection on the 1st, 2nd, 3rd, 5th, 7th and 9th day of the experiment. The assessment was carried out for (**A**) 10 min and (**B**) 30 min. The values represent mean ± SEM (*n* = 8 animals per group). ^^^^ *p* < 0.0001 versus saline on the 1st day; *** *p* < 0.001, ** *p* < 0.01, * *p* < 0.05 versus mephedrone on the 1st day (Bonferroni’s post-hoc test).

As was demonstrated in Figure 3, an acute administration of mephedrone (5 mg/kg) on the first day of the experiment increased locomotor activity of the tested animals as compared to the saline-treated group. After the second dose of mephedrone (5 mg/kg, which was injected on the second day of the study), mice were significantly less active than after the first dose. Their locomotion was comparable to the one detected for the saline-treated group. The development of tolerance to the mephedrone-induced hyperlocomotion was also observed on the next days of our experiment (i.e., on the third, fifth and seventh days). The same behavioral pattern was detected by a 10 min and a 30 min measurement, though differences between the results obtained for the mephedrone-treated group on the first day and on other days were more pronounced when the locomotor activity was assessed for 30 min. Two-way ANOVA indicated a significant treatment-time period interaction [F(4,70) = 2.60; *p* = 0.0433] with a significant effect of the introduced treatment [F(1,70) = 37.50, *p* < 0.0001] and a significant effect of time period [F(4,70) = 4.55, *p* = 0.0025] for the 10 min measurement, as well as a significant treatment-time period interaction [F(4,70)=5.41; *p* = 0.0007] with a significant effect of the introduced treatment [F(1,70) = 22.86, *p* < 0.0001] and a significant effect of time period [F(4,70) = 5.71, *p* = 0.0005] for the 30 min measurement.

On the ninth day, when all animals received only saline, there were no differences between the spontaneous locomotor activity recorded for both tested groups (*p* > 0.05).

### 3.3. Effects of Mephedrone in the Rotarod Test and in the Chimney Test in Mice

An acute administration of mephedrone at the tested doses (i.e., 0.05, 0.125, 0.25, 0.5, and 1 mg/kg) did not influence mice behavior in the rotarod test [one-way ANOVA: F(5,42) = 1.106; *p* = 0.3719] and in the chimney test [one-way ANOVA: F(5,42) = 0.3461; *p* = 0.8819]. Animals given the drug spent the same duration of time on the rotating rod and in the chimney as the saline-treated mice did (Table 1).

### 3.4. Effects of Mephedrone in the EPM Test in Mice

**Figure 4:**
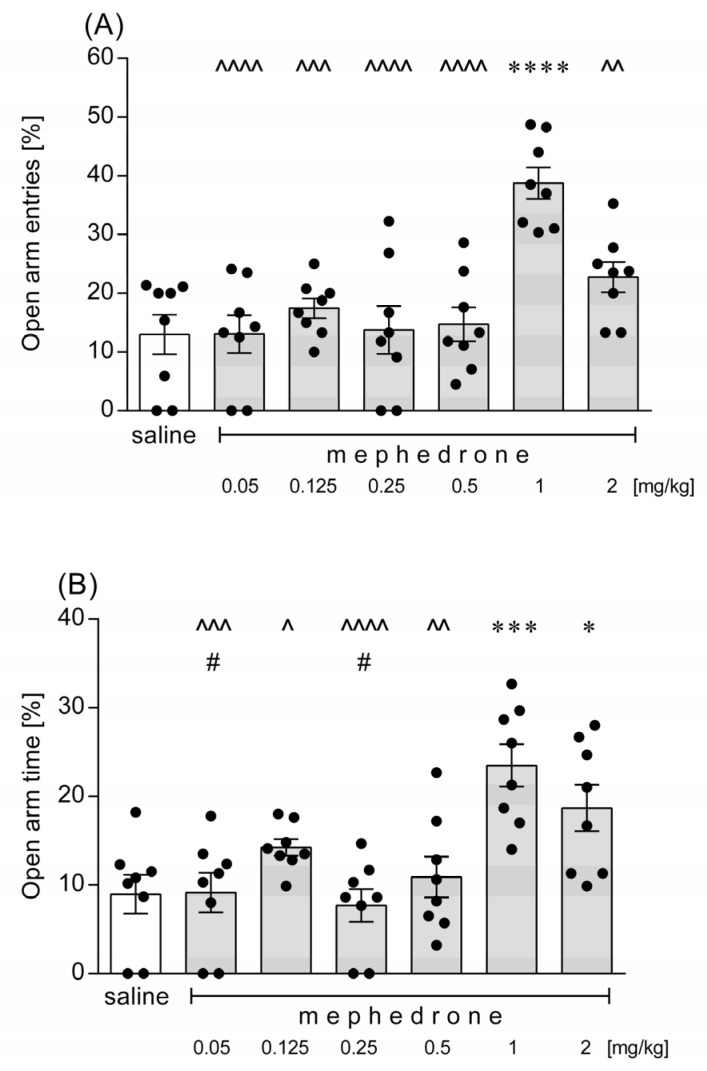
Effects of an acute administration of mephedrone in the elevated plus maze in mice. A single dose of mephedrone (0.05, 0.125, 0.25, 0.5, 1 or 2 mg/kg) or saline was given intraperitoneally 20 min before the behavioral test. (**A**) open arm entries and (**B**) the time spent in open arms were measured for each animal. The values represent mean ± SEM (*n* = 8 mice per group). **** *p* < 0.0001, *** *p* < 0.001, * *p* < 0.05 versus saline; ^^^^ *p* < 0.0001, ^^^ *p* < 0.001, ^^ *p* < 0.01, ^ *p* < 0.05 versus mephedrone 1 mg/kg; # *p* < 0.05 versus mephedrone 2 mg/kg (Tukey’s post-hoc test).

Mephedrone given at a dose of 1 mg/kg increased the percentage of entries into the open arms of the EPM in mice. Other tested doses of the drug (i.e., 0.05, 0.125, 0.25, 0.5 and 2.0 mg/kg) did not influence this parameter (Figure 4A). One-way ANOVA demonstrated significant differences between the compared groups: [F(6,49) = 9.896; *p* < 0.0001]. An acute administration of mephedrone at a dose of 1 or 2 mg/kg also prolonged the time spent in the open arms of the maze, and this effect was more pronounced for the dose of 1 mg/kg. Lower tested doses of the drug did not affect behavior of mice in the open arms (Figure 4B). One-way ANOVA revealed the following results: [F(6,49) = 7.641; *p* < 0.0001]. None of the tested doses of mephedrone changed the total number of arm entries (*p* > 0.05; data not shown).

### 3.5. Effects of Mephedrone on the PTZ-Induced Seizures in Mice

PTZ produced clonic seizures in all tested mice, whereas tonic seizures and death were recorded for 8/10 animals. An acute administration of mephedrone at the tested doses (i.e., 1.25, 2.5 and 5 mg/kg) did not protect mice from the PTZ-induced clonic seizures, tonic seizures, or mortality (*p* > 0.05), which was summarized in Table 2.

### 3.6. Effects of Mephedrone in the mEMP Test in Mice

**Figure 5:**
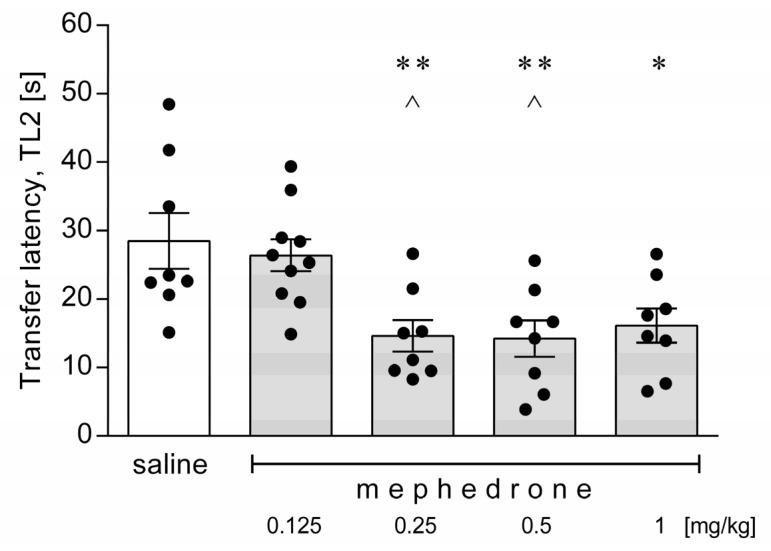
Effects of an acute administration of mephedrone in the modified elevated plus maze (mEPM)in mice. A single dose of mephedrone (0.125, 0.25, 0.5 or 1 mg/kg) or saline was given intraperitoneally 20 min before the behavioral test. The transfer latency during the retention session (TL2) was measured for each animal. The values represent mean ± SEM (*n* = 8–10 mice per group). ** *p* < 0.01, * *p* < 0.05 versus saline; ^ *p* < 0.05 versus mephedrone 0.125 mg/kg (Tukey’s post-hoc test).

An acute administration of mephedrone (0.125, 0.25, 0.5 and 1 mg/kg) did not influence the TL1 values obtained in the acquisition session (*p* > 0.05; data not shown). However, during the retention session, animals given mephedrone at a dose of 0.25, 0.5 or 1 mg/kg reached one of the enclosed arms more quickly than the saline-treated mice, which was recorded as a shorter transfer latency (TL2) (Figure 5). Mice subjected to a dose of 0.125 mg/kg behaved in the mEMP similarly to the control animals (*p* > 0.05). One-way ANOVA demonstrated the following results: [F(6,37) = 6.018; *p* = 0.0008].

### 3.7. Effects of Mephedrone in the NOR Test in Mice

As given in Table 3, none of the acute tested doses of mephedrone (i.e., 0.125, 0.25, 0.5 and 1 mg/kg) affected the behavior of animals in the NOR test in mice. PI values calculated for the mephedrone-treated animals were similar to the ones obtained for the control group. One-way ANOVA confirmed a lack of significant differences between the compared groups: [F(4,35) = 0.5456; *p* = 0.7034].

## 4. Discussion

### 4.1. Effects of Mephedrone on the Spontaneous Locomotor Activity and Tolerance to the Mephedrone-Induced Hyperlocomotion

As expected, in our study, mephedrone (a drug popular during dance parties) given acutely via an intraperitoneal injection induced hyperactivity in mice in a dose-dependent manner. The active dose of mephedrone was 5 mg/kg, whereas ineffective doses were between 0.05 and 2.5 mg/kg. These outcomes are in line with findings of other authors who had demonstrated that mephedrone in a dose range of 3 to 40 mg/kg increased spontaneous locomotion of various murine [22,33,52] and rat [12,17,20,30,34] strains, following an intraperitoneal [30,33,37], intravenous [12], oral [12], and subcutaneous [17,20,34] administration as well as after an acute [20,33,34], repeated "binge-style" [17,37,52], and intermittent exposure [30]. Increased ambulatory activation that followed mephedrone treatment was observed in both adult [12,37] and adolescent rodents [6]. However, according to literature data, some strain-specific responses can be detected. E.g., Wright et al. [34] found out that Sprague-Dawley rats responded to the mephedrone stimulatory effects more profoundly than Wistar rats. According to the literature data [17,20], the intensity of the mephedrone-induced hyperlocomotion can be comparable to the one observed after the same dose of MDMA, but it is milder than the one detected after amphetamine. The functional observational battery (FOB) carried out by Marusich et al. [33] in ICR mice revealed that the mephedrone-related activation of the spontaneous locomotion in animals may be accompanied by general stimulation (i.e., tense body, sudden darting), increased stereotyped head weaving and increased head circling. However, based on the study by Huang et al. [53], it seems that mephedrone does not always increase locomotor activity. The authors demonstrated a dose-dependent decrease in wheel running in Wistar rats after exposure to mephedrone at a dose ≥5.6 mg/kg. Such a response of animals was similar to the one observed after treatment with MDMA (≥5.6 mg/kg). On the other hand, the mephedrone-induced ambulatory hyperactivity seems to be irrespective of the circadian cycle [6,30,39,40,41,54] as well as independent of the ambient temperature [34].

Our further results confirmed that mephedrone easily crosses the blood-brain barrier and peaks in the brain quickly after exposure since the most pronounced effects on the spontaneous locomotor activity were observed within the following 10 min. We also demonstrated that these effects were transient, and they lasted for a relatively short time; the motor activity of the tested mice returned to the control values 50 min after the injection (5 mg/kg). Similar outcomes were obtained by other authors, e.g., Kehr et al. [20] and Shortall et al. [30], who reported that the mephedrone-induced ambulatory hyperactivity is not a long-term one and it disappears earlier than motor stimulation detected after amphetamine and MDMA exposure, respectively. These observations are in line with data obtained from the human population, which suggests that mephedrone is a substance with quick metabolism and short half-lives [8]. Mephedrone users state that the onset of desired effects of the drug usually appears within 15–45 min after oral ingestion and a few min after nasal insufflation, but they are short lived and fade within 2–3 h. Moreover, the duration of the desired effects after intravenous administration of mephedrone is even shorter, and it lasts approximately 30 min [9,55]. This explains why mephedrone users readily re-dose the drug, similarly as it is observed for cocaine [13,56].

Most probably, the mephedrone-induced increase in mobility is due to alterations in the serotonergic and dopaminergic neurotransmissions since antagonism of the serotonin 5-HT_1A_, 5-HT_1B_, and 5-HT_2_ receptors with WAY-100635, GR 127935 [37], and ketanserin [22] respectively, inhibition of serotonin synthesis with para-chlorophenylalanine [22], 5,7-dihydroxytryptamine-induced serotonin depletion [37] as well as pre-treatment with a non-selective antagonist of dopamine receptors; haloperidol [22] and selective blockage of D_1_ receptors with SCH23390 [41,57] prevented mephedrone-induced hyperlocomotion. Interestingly, sulpiride (i.e., an antagonist of dopamine D_2_ receptor) potentiated the mephedrone-induced increase in ambulatory activity [41], whereas neither 6-hydroxytryamine (i.e., a neurotoxin causing dopamine depletion) nor SB-258719 (an antagonist of the 5-HT_7_ receptors) affected effects of mephedrone [37]. Mayer et al. [58] demonstrated that bioactive metabolites of mephedrone (e.g., nor-mephedrone) contribute to behavioral stimulatory effects of the drug. Kehr et al. [20] reported that 20 min after administration of a low mephedrone dose (1 mg/kg), serotonin and dopamine levels in the brain of Sprague-Dawley rats were significantly increased.

Another set of our experiments showed that administration of mephedrone (5 mg/kg) for several days may result in the development of tolerance to the mephedrone-related ambulatory hyperactivity. According to medical literature, drug tolerance is defined as a gradual decrease in the effect of a given agent when it is administered repeatedly [59]. Actually, the second intraperitoneal dose of mephedrone (given 24 h after the first one) did not increase animals’ mobility, and the same trend was observed after subsequent injections. However, we did not detect the development of withdrawal effects after discontinuation of mephedrone treatment. As far as we know, there are only a few reports about the development of tolerance to mephedrone-induced effects in animals (e.g., the work of [36,37,60]). According to the literature data [61], tolerance to mephedrone effects after a long-term consumption may also be detected in humans. Tough, in the study by Shortall and colleagues [37], the ”binge-style“ mephedrone administration (several doses in one session, i.e., three times with a 2 h interval, which mirrors mephedrone use by humans) caused reproducible hyperactivity after each dose (without any signs of tolerance to the drug), with a quick onset and short duration.

Tolerance to the central effects of other psychostimulants (e.g., MDMA, amphetamine, or cocaine) is a widely described phenomenon [62,63,64,65], but little is known about the potential development of tolerance to effects induced by mephedrone. Furthermore, the development of tolerance to the effects of different cathinone stereoisomers has been observed in preclinical studies as well [66,67,68]. Determination of mechanisms/pathways involved in the development of tolerance to mephedrone-induced hyperlocomotion could cast a new light on dependence and/or addiction to this drug. So far, it is suggested that the dopaminergic system [36] and endogenous kappa opioid pathways [69] are implicated in the development of tolerance to mephedrone activity. Most probably, other neurotransmissions contribute to this process as well.

### 4.2. Effects of Mephedrone on Motor Coordination

The rotarod and the chimney tests are two widely recognized behavioral paradigms that are used in preclinical studies to evaluate the ability of a given drug to interfere with motor coordination in rodents. Motor impairment is indicated by the decreased time spent on the rotarod or by the inability of mice to climb backward up the tube within 60 min. Additionally, the rotarod test assesses the sense of balance [70,71], whereas the chimney estimates an impact of the introduced treatment on muscle relaxation [71]. In consequence, when motor incoordination is observed only in the rotarod test, it can be assumed that a given drug exerts depressive effects on the central nervous system. However, when motor incoordination is observed in both tests, most probably, a given drug has a myorelaxant potential [71,72,73]. In the present study, none of the tested mephedrone doses (i.e., 0.05–1 mg/kg) disturbed motor coordination of animals or induced myorelaxation. It was in line with outcomes reported by Marusich et al. [33], who also found out that mephedrone within the tested dose range of 3–56 mg/kg (i.p.) did not affect behavior of ICR mice on the rotarod.

### 4.3. Effects of Mephedrone in the EPM Test

The EMP test is used to evaluate an anxiogenic/anxiolytic potential of a given drug in preclinical studies carried out in rodents. Animals’ behavior in this task is based on an innate conflict between the preference for safe, protected places (i.e., closed arms in the case of the EPM) and natural motivation to explore novel areas even though they are potentially uncomfortable (i.e., open arms in the case of the EPM). Thus, the anxiogenic-like effect is indicated by a decreased number of entries to the open arms of the maze and by the shorter time spent in these arms. On the other hand, the anxiolytic-like effect is demonstrated by an increased number of entries to the open arms and by the prolonged time spent in these arms [46]. In the present study, mephedrone displayed the anxiolytic dose-dependent potential. The two highest doses of the drug tested in the experiment (i.e., 1 and 2 mg/kg) significantly lengthened the time spent by animals in the open arms, whereas only 1 mg/kg of mephedrone significantly increased the percentage of open arm entries. Our results are contrary to the reports by Pail and colleagues [32] or den Hollander and colleagues [35], who demonstrated the anxiogenic potential of mephedrone or no effect of mephedrone in the EPM in mice. Most probably, different experimental designs, murine strains, tested doses, and/or laboratory conditions are responsible for these discrepancies in the observed mephedrone activity. Similarly, Sichova et al. [74] noted bi-directional (i.e., anxiogenic and anxiolytic) effects of mephedrone in the open field test in Wistar rats, which were dependent on the applied dose. Increased exploration in the central zones, suggesting a decreased level of anxiety, was observed after lower mephedrone doses (2.5 and 5 mg/kg, s.c.), whereas more pronounced thigmotaxis, suggesting an elevated level of anxiety, was noted following the highest tested dose of the drug (i.e., 20 mg/kg). In our experiments, low doses of mephedrone were applied. The thigmotaxic effect of mephedrone (15 and 30 mg/kg, i.p.) in Wistar rats was also recorded by Motbey et al. [6]. In fact, anxiety is one of the adverse reactions reported after mephedrone use in humans [75].

### 4.4. Effects of Mephedrone in the PTZ Seizure Model

In the present study, the PTZ seizure model was used to evaluate an anticonvulsant or proconvulsant potential of mephedrone. An acute systemic administration of PTZ is usually applied in preclinical experiments for rapid screening of antiepileptic drugs. Such exposure to PTZ induces both clonic and tonic seizures, and it may result in the death of the tested animals [50,76]. None of the mephedrone doses used in our study (i.e., 1.25, 2.5 and 5 mg/kg) protected mice from the PTZ-induced clonic seizures, tonic seizures, or mortality. On the other hand, the obtained results did not suggest the proconvulsant potential of mephedrone at the tested doses, which was in line with observations made by Marusich and colleagues [33]. In the cited study carried out in ICR mice, this cathinone did not produce tremors or convulsions even though it was administered i.p. at much higher doses (up to 56 mg/kg) than the ones applied by us. Human-based research on the neurotoxic effects is scarce, but according to the published records [62,64], tremors, convulsions, and self-limiting pre-hospital seizures have been mentioned among the mephedrone-related adverse reactions. The set of experiments carried out by Martinez-Clemente et al. [29] demonstrated that mephedrone is able to induce some transient dopaminergic and serotoninergic neurotoxicity, but there is a significant body of evidence [17,35,52] to indicate that these effects do not occur frequently and that they are not long-term when mephedrone is used alone. However, this cathinone may amplify the toxic effects of other illicit substances toward the monoaminergic systems when taken concurrently [28].

### 4.5. Effects of Mephedrone on Various Aspects of Learning and Memory

In order to evaluate the impact of mephedrone use on learning and memory, we applied the mEPM and the NOR tests. Both tests are widely used to investigate the influence of different compounds with psychotropic activity on memory/learning processes [17,77,78]. The first test, the mEPM test, is a simple paradigm measuring spatial memory. As mentioned above, it is natural for rodents to avoid open and elevated spaces, so they escape from the open arms of the maze to the enclosed ones. The time period needed for a given animal to move from an open arm to an enclosed arm, which is called “transfer latency”, is used as an index of learning and memory processes. During the experiment, two sessions, with a 24 h interval, are carried out, i.e., the acquisition session and the retention session. Prolongation of transfer latency assessed at the retention session is treated as a sign of memory impairments in a tested animal. It suggests that the administered substance exerts amnestic effects since the animal does not remember the configuration of arms in the maze. On the other hand, shortening of transfer latency during the retention session indicates that a given drug improves memory in animals [79]. As for the second test, the NOR test, it evaluates visual recognition memory (particularly, object recognition memory). An innate preference for novelty in rodents is used here. During the experiment, three sessions are carried out, i.e., the habituation session, acquisition session, and trial session. At the acquisition session, a given animal is allowed to explore two identical objects, and at the trial session, which takes place 1 h after the acquisition session, one of the objects is replaced with a novel object. If an animal remembers the familiar object, it spends most of the time exploring a new one. There are several indexes measuring cognitive functions in the NOR, including the PI used in the present study. The PI above 50% indicates animal’s preference for a novel object, whereas the PI below 50% indicates animal’s preference for a familiar object. The IP about 50% demonstrates no preference for any object [78,80].

In our experiments, mephedrone was administered 20 min before the acquisition session (in both tests), and the effects of this cathinone on the acquisition of memory were investigated at the retention/trial session. Outcomes obtained from the mEPM and the NOR tests suggest that mephedrone (at least at the tested doses, i.e., 0.125–1 mg/kg) does not affect recognition memory, but it improves spatial memory in a dose-dependent manner. The higher tested doses (0.25–1 mg/kg) of the drug induced significant shortening of transfer latency during the retention session in the mEPM, whereas the lowest applied dose (0.125 mg/kg) was ineffective. Our observations are on the contrary to the ones described by Motbey et al. [39], who highlighted the negative effects of mephedrone administration on novel object recognition. However, the above-mentioned experiments were differently designed than ours, i.e., Wistar rats were given mephedrone at a dose of 30 mg/kg/day for 10 days, and the behavioral assessment was made 5 weeks of drug abstinence. Thus, in comparison to our study, the tested dose was much higher, the drug was given for several days instead of an acute administration, and the NOR test was carried out 35 days after exposure to the drug. Moreover, Motbey and colleagues [39] used adolescent Wistar rats, whereas adult albino Swiss mice were used in our study. According to literature data as well as to the results of the present study, it is probable that mephedrone differently affects various types of memory in distinct laboratory animals. Den Hollander et al. [35] reported that mephedrone reduces working memory performance in the T-maze in C57BL/J6 mice, Shortall and others [30] noted that it also impairs retention of fear motivated contextual memory in Lister hooded rats, whereas Wright and colleagues [34] found out that this cathinone may improve visuospatial associative memory in rhesus macaques, but it does not influence spatial working memory. Human-based research has indicated that mephedrone use may impair acute working memory and cognitive performance [81,82]. Loss of concentration and memory loss are listed among adverse reactions related to the central nervous system that may occur after mephedrone use [75]. Most probably, MMP-2 and MMP-9 present in different brain regions are implicated in mephedrone’s activity toward memory processes [26,83]. Mephedrone effects on learning are inconclusive, and they depend on various factors, including the age of a tested animal, its species, dosing schedule. Naseri et al. [27] showed that offspring born from mothers repeatedly exposed to mephedrone during pregnancy demonstrated impairments in spatial learning and reference memory in the Morris water maze. Similarly, rats subjected to mephedrone treatment during late adolescence showed impairments in spatial learning in Barnes maze task in adulthood [26]. However, results of other studies suggested that effects of mephedrone on spatial learning and memory (Morris water maze) in adolescent rodents are limited [36]. Furthermore, in experiments by den Holleander et al. [35] C57BL/J6 mice treated with mephedrone better performed than the control mice in finding a platform hidden under water in Morris water maze, but the mechanism behind these observations has not been explained.

## 5. Conclusions

In conclusion, the major findings of the current study contribute to the general knowledge about the central effects of mephedrone. We showed that an acute administration of this drug in adult albino Swiss mice (a) does not affect motor coordination, (b) may have an anxiolytic potential, (c) does not produce anticonvulsant or proconvulsant effects, and (d) does not affect recognition memory, but it may improve spatial memory. Moreover, we demonstrated that the mephedrone-induced ambulatory hyperactivity is rapid and short lived and that the repeated administration of this cathinone may lead to the development of tolerance to these stimulatory effects. Discrepancies between the outcomes of our study and observations made by other authors in relation to the EMP and the NOR test may result from using distinct animal strains and tested doses as well as from differences in laboratory conditions, project designs, or duration of experiments.

## Figures and Tables

**Table 1:** Effects of an acute administration of mephedrone on motor coordination in mice.

Substance (mg/kg)	Time on the Rotarod (s)	Time in the Chimney (s)
Saline	55.05 ± 4.500	6.580 ± 1.050
Mephedrone 0.05	53.10 ± 3.600	6.530 ± 1.400
Mephedrone 0.125	46.60 ± 5.600	7.780 ± 1.700
Mephedrone 0.25	43.68 ± 5.600	5.990 ± 0.960
Mephedrone 0.5	53.83 ± 4.300	7.420 ± 1.300
Mephedrone 1.0	55.16 ± 3.800	5.970 ± 1.010

A single dose of mephedrone (0.05, 0.125, 0.25, 0.5 or 1 mg/kg) or saline was given intraperitoneally 20 min before the behavioral test. The time spent on the rotarod and the time spent in the chimney were measured for each animal. The values represent mean ± SEM (*n* = 8 mice per group) (Tukey’s post-hoc test).

**Table 2:** Effects of mephedrone on the PTZ-induced seizures in mice.

Substance (mg/kg)	Number of Mice with Clonic Seizures	Number of Mice with Tonic Seizures	Mortality
Saline	0/10	0/10	0/10
Saline + PTZ 110	10/10	8/10	8/10
Mephedrone 1.25 + PTZ 110	10/10	8/10	7/10
Mephedrone 2.5 + PTZ 110	9/10	8/10	7/10
Mephedrone 5.0 + PTZ 110	9/10	7/10	7/10

A single injection of mephedrone (1.25, 2.5, 5 mg/kg) or saline was administered intraperitoneally 20 min before subcutaneous injection of pentylenetetrazole (PTZ, 110 mg/kg). Immediately after treatment with PTZ, animals were observed for 60 min for the incidents of clonic seizures, tonic convulsions, and death. *n* = 10 mice per group (Kruskal–Wallis’s post-hoc test).

**Table 3:** Effects of an acute administration of mephedrone in the novel object recognition (NOR) test in mice.

Substance (mg/kg)	Preference Index (PI)
Saline	41.42 ± 2.358
Mephedrone 0.125	39.81 ± 1.747
Mephedrone 0.25	39.34 ± 3.359
Mephedrone 0.5	36.17 ± 3.404
Mephedrone 1.0	37.62 ± 2.514

A single dose of mephedrone (0.125, 0.25, 0.5 or 1 mg/kg) or saline was given intraperitoneally 20 min before the behavioral test. The preference index (PI) was calculated for each animal. The values represent mean ± SEM (*n* = 8–10 mice per group) (Tukey’s post-hoc test).

## Data Availability

The data presented in this study are available on request from the corresponding author.
